# Software testing in microbial bioinformatics: a call to action

**DOI:** 10.1099/mgen.0.000790

**Published:** 2022-03-08

**Authors:** Boas C.L. van der Putten, C. I. Mendes, Brooke M. Talbot, Jolinda de Korne-Elenbaas, Rafael Mamede, Pedro Vila-Cerqueira, Luis Pedro Coelho, Christopher A. Gulvik, Lee S. Katz, The ASM NGS 2020 Hackathon participants

**Affiliations:** ^1^​ Department of Medical Microbiology, Amsterdam UMC, University of Amsterdam, the Netherlands; ^2^​ Department of Global Health, Amsterdam Institute for Global Health and Development, Amsterdam UMC, University of Amsterdam, the Netherlands; ^3^​ Instituto de Microbiologia, Instituto de Medicina Molecular, Faculdade de Medicina, Universidade de Lisboa, Lisboa, Portugal; ^4^​ Department of Biological and Biomedical Sciences, Emory University, Atlanta, GA, USA; ^5^​ Department of Infectious Diseases, Public Health Laboratory, Public Health Service of Amsterdam, the Netherlands; ^6^​ Institute of Science and Technology for Brain-Inspired Intelligence, Fudan University, PR China; ^7^​ Key Laboratory of Computational Neuroscience and Brain-Inspired Intelligence, PR China; ^8^​ Bacterial Special Pathogens Branch, Division of High-Consequence Pathogens and Pathology, Centers for Disease Control and Prevention, Atlanta, GA, USA; ^9^​ Enteric Diseases Laboratory Branch, Division of Foodborne, Waterborne, and Environmental Diseases, Centers for Disease Control and Prevention, Atlanta, GA, USA; ^10^​ Center for Food Safety, University of Georgia, Griffin, GA, USA

**Keywords:** software testing, continuous integration, computational biology

## Abstract

Computational algorithms have become an essential component of research, with great efforts by the scientific community to raise standards on development and distribution of code. Despite these efforts, sustainability and reproducibility are major issues since continued validation through software testing is still not a widely adopted practice. Here, we report seven recommendations that help researchers implement software testing in microbial bioinformatics. We have developed these recommendations based on our experience from a collaborative hackathon organised prior to the American Society for Microbiology Next Generation Sequencing (ASM NGS) 2020 conference. We also present a repository hosting examples and guidelines for testing, available from https://github.com/microbinfie-hackathon2020/CSIS.

## Impact Statement

In the field of microbial bioinformatics, good software engineering practises are not yet widely adopted. Many microbial bioinformaticians start out as (micro)biologists and subsequently learn how to code. Without abundant formal training, a lot of education about good software engineering practices comes down to an exchange of information within the microbial bioinformatics community. This paper serves as a resource that could help microbial bioinformaticians get started with software testing if they have not had formal training.

## Background

Computational algorithms, software, and workflows have enhanced the breadth and depth of microbiological research and expanded the capacity of infectious disease surveillance in public health practice. Scientists now have a wealth of bioinformatic tools for addressing pertinent questions quickly and keeping pace with the availability of larger and more complex biological datasets. Despite these advances, we are finding ourselves in a crisis of computational reproducibility [1].

**Fig. 1.**
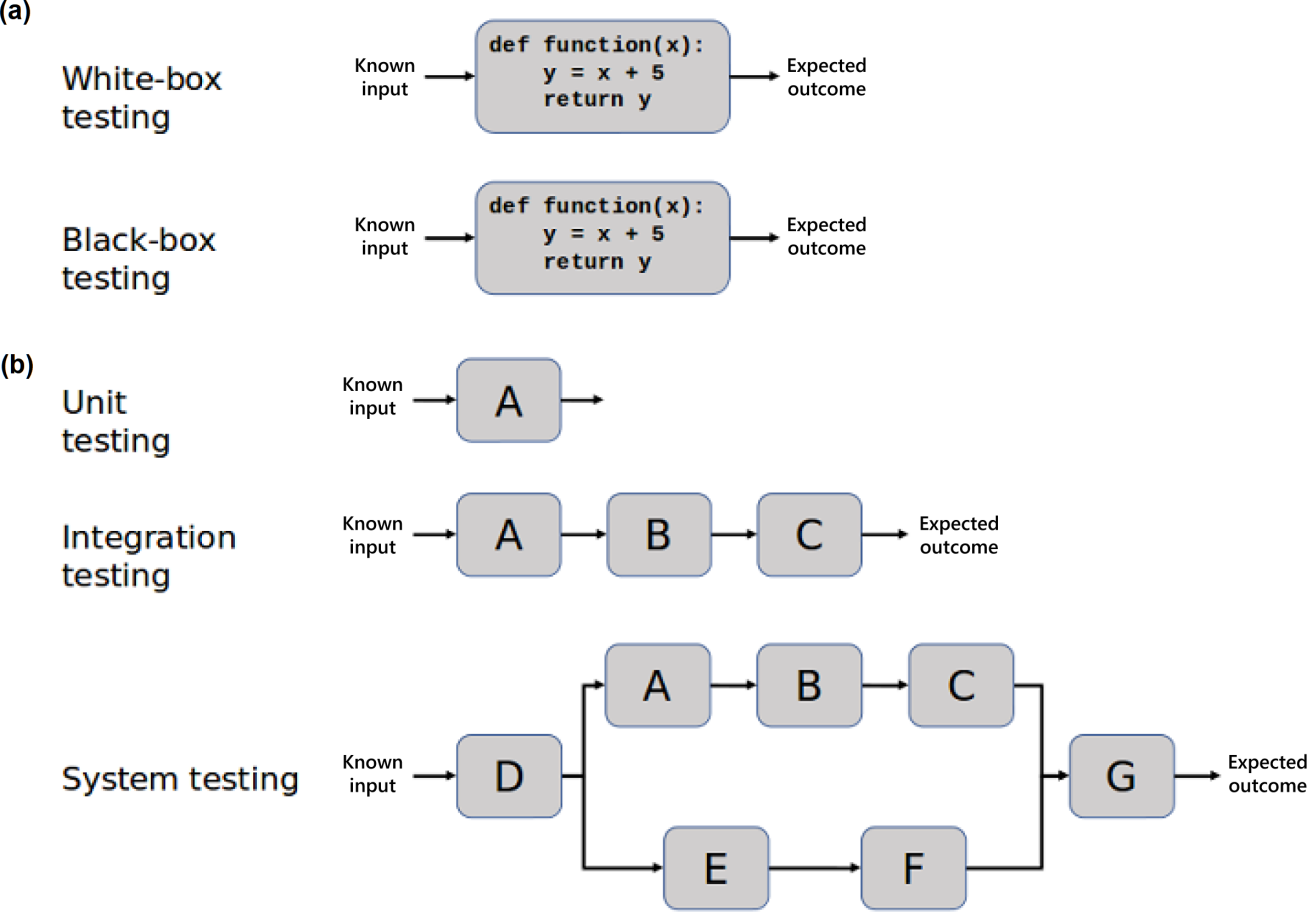
Testing strategies. (**a**) White-box vs. black-box testing. In white-box testing, the tester knows the underlying code and structure of the software, where the tester does not know this in black-box testing. Note that this distinction is not strictly dichotomous and is considered less useful nowadays (b) Unit vs. integration vs. system testing. When software comprises several modules, it is possible to test each single module (unit testing), groups of related modules (integration testing) or all modules (system testing). Note that the terms white-box testing and unit testing are sometimes used interchangeably but relate to different concepts.

Modern software engineering advocates reliable software testing standards and best practices. Different approaches are employed: from unit testing to system testing [2], going from testing every individual component to testing a tool as a whole (Fig. 1). The extent of testing is a balance between the resources available and increasing sustainability and reproducibility. Continuous Integration (CI), where code changes are frequently integrated and assertion of the new code’s correctness before integration is often automatedly performed through tests, provides a robust approach for ensuring the reproducibility of scientific results without requiring human interaction. Comprehensive testing of scientific software might prevent computational errors which subsequently lead to erroneous results and retractions [3, 4]. However, the role of testing extends beyond that, as it also provides a way to measure software coverage, and therefore its robustness, allowing for reported issues to be converted into testable actions (regression tests), and the expansion and refactoring of existing code without compromising its function.

Software testing among peers across fields aligns with previous efforts of hackathons to create a more unified and informed bioinformatics software community [5]. In this context, we hosted a cooperative hackathon prior to the ASM NGS conference in 2020, demonstrating that the microbial bioinformatics community can contribute to software sustainability using a collaborative platform (Table S1, available in the online version of this article). From this experience, we would like to propose collaborative software testing as an opportunity to continuously engage software users, developers, and students to unify scientific work across domains. We have outlined the following recommendations for ensuring software sustainability through testing and offer a repository of automated test knowledge and examples at the Code Safety Inspection Service (CSIS) repository on GitHub (https://github.com/microbinfie-hackathon2020/CSIS).

## Recommendations

Based on our experiences from the ASM NGS 2020 hackathon, we developed seven recommendations that can be followed during software development.

### Establish software needs and testing goals

Manually testing the functionality of a tool is feasible in early development but can become laborious as the software matures. Developers may establish software needs and testing goals during the planning and designing stages to ensure an efficient testing structure. Table 1 provides an overview of testing methodologies and can serve as a guide to developers that aim to implement testing practises. A minimal test set could address the validation of core components or the programme as a whole (system testing) and gradually progress toward verification of key functions which can accommodate code changes over time (unit testing, Fig. 1). Ideally, testing should be implemented from the early stages of software development (test-driven development). Defining the scope of testing is important before developing tests. For pipeline development, testing of each individual component can be laborious and can be expedited if those components already implement testing of their own. Testing of the pipeline itself should take priority.

**Table 1.** Overview of testing approaches. Software testing can be separated into three types: installation, functionality and destructive. Each component is described, followed by an example on a real-life application on *Software X*, a hypothetical nucleotide sequence annotation tool

Name	Description	Example
**Installation testing: can the software be invoked on different setups?**		
**Installation testing**	Can the software be installed on different platforms?	*Test whether Software X can be installed using apt-get, pip, conda and from source*.
**Configuration testing**	With which dependencies can the software be used?	*Test whether Software X can be used with different versions of blast+*.
**Implementation testing**	Do different implementations work similarly enough?	*Test whether Software X works the same between the standalone and webserver versions*.
**Compatibility testing**	Are newer versions compatible with previous input/output?	*Test whether Software X can be used with older versions of the UniProtKB database*.
**Static testing**	Is the source code syntactically correct?	*Check whether all opening braces have corresponding closing braces or whether code is indented correctly in Software X*.
**Standard functionality testing: does the software do what it should in daily use?**		
**Use case testing**	Can the software do what it is supposed to do regularly?	*Test whether Software X can annotate different FASTA files: with spaces in the header, without a header, an empty file, with spaces in the sequence, with unknown characters in the sequences, et cetera*.
**Workflow testing**	Can the software successfully traverse each path in the analysis?	*Test whether Software X works in different modes (using fast mode or using one dependency over the other*).
**Sanity testing**	Can the software be invoked without errors?	*Test whether Software X works correctly without flags, or when checking dependencies or displaying help info*.
**Destructive testing: what makes the software fail?**		
**Mutation testing**	How do the current tests handle harmful alterations to the software?	*Test whether changing a single addition to a subtraction within Software X causes the test suite to fail*.
**Load testing**	At what input size does the software fail?	*Test whether Software X can annotate a small plasmid (10 kbp), a medium-size genome (2 Mbp) or an unrealistically large genome for a prokaryote (1 Gbp*).
**Fault injection**	Does the software fail if faults are introduced and how is this handled?	*Test whether Software X fails if nonsense functions are introduced in the gene calling code*.

Gbp, Giga-base-pair; kbp, kilo-base-pair; Mbp, Mega-base-pair.

### Input test files: the good, the bad, and the ugly

When testing, it is important to include test files with known expected outcomes for a successful run. However, it is equally important to include files or other inputs on which the tool is expected to fail. For example, some tools should recognize and report an empty input file or a wrong input format. Therefore, the test dataset should be small enough to be easily deployed (see recommendation #4) but as large as necessary to cover all intended test cases. Data provenance should be disclosed, either if it’s from real data or originated *in silico*. Typically, a small test data is packaged with the software. Examples of valid and invalid file formats are available through the BioJulia project (https://github.com/BioJulia/BioFmtSpecimens). The nf-core project (https://nf-co.re/) provides a repository with test data for a myriad of cases (https://github.com/nf-core/test-datasets).

### Use an established framework to implement testing

Understanding the test workflow can not only ensure continued software development but also the integrity of the project for developers and users. Testing frameworks improve test development and efficiency. Examples include unittest (https://docs.python.org/3/library/unittest.html) or pytest (https://docs.pytest.org/en/stable/) for Python, and testthat (https://testthat.r-lib.org/) for R, testing interfaces such as TAP (http://testanything.org/), or built-in test attributes such as in Rust. Although many tests can be implemented using a combination of frameworks, personal preferences (e.g. amount of boilerplate code required) might drive your choice. Additionally, in Github Actions the formulas of each test block can be explicitly stated using the standardised and easy-to-follow YAML (https://yaml.org/, Fig. S1, available in the online version of this article), already adopted by most continuous integration platforms (recommendation #4). For containerised software, testing considerations differ slightly and have been covered previously by Gruening *et al*. (2019) [6].

### Testing is good, automated testing is better

When designing tests, planning for automation saves development time. Whether your tests are small or comprehensive, automatic triggering of tests will help reduce your workload. Many platforms trigger tests automatically based on a set of user-defined conditions. Platforms such as GitHub Actions (https://github.com/features/actions) and GitLab CI (https://about.gitlab.com/stages-devops-lifecycle/continuous-integration) offer straightforward automated testing of code seamlessly upon deployment. A typical workflow, consisting of a minimal testing framework (see recommendation #1 and #3) and a small test dataset (see recommendation #2), can then be directly integrated within your project hosted on a version control system, such as GitHub (https://github.com/), and directly integrated with a continuous integration provider, such as GitHub Actions in GitHub. Testing considerations for containerised software has been covered previously by Gruening *et al*. (2019) [6].

### Ensure portability by testing on several platforms

The result of an automated test in the context of one computational workspace does not ensure the same result will be obtained in a different setup. It is important to ensure your software can be installed and used across supported platforms. One way to ensure this is to test on different environments, with varying dependency versions (e.g. multiple Python versions, instead of only the most recent one). Developers can gain increased benefits of testing if tests are run on different setups automatically (see recommendation #4 and Fig. S1).

### Showcase the tests

For prospective users, it is good to know whether you have tested your software and, if so, which tests you have included. This can be done by displaying a badge [7] (see https://github.com/microbinfie-hackathon2020/CSIS/blob/main/README.md#example-software-testing), or linking to your defined testing strategy e.g. a GitHub Actions YAML, (see recommendation #2, Fig. S1). Documenting the testing goal and process enables end-users to easily check tool functionality and the level of testing [8].

It may be helpful to contact the authors, directly or through issues in the code repository, whose software you have tested to share successful outcomes or if you encountered abnormal behaviour or component failures. An external perspective can be useful to find bugs that the authors are unaware of. A set of issue templates for various situations is available in the CSIS repository on GitHub (https://github.com/microbinfie-hackathon2020/CSIS/tree/main/templates).

### Encourage others to test your software

Software testing can be crowdsourced, as showcased by the ASM NGS 2020 hackathon. Software suites such as Pangolin (https://github.com/cov-lineages/pangolin) [9] and chewBBACA (https://github.com/B-UMMI/chewBBACA) [10] have implemented automated testing developed during the hackathon. For developers, crowdsourcing offers the benefits of fresh eyes on your software. Feedback and contributions from users can expedite the implementation of software testing practices. It also contributes to software sustainability by creating community buy-in, which ultimately helps the software maintainers keep pace with dependency changes and identify current user needs.

## Conclusions

Testing is a critical aspect of scientific software development, but automated software testing remains underused in scientific software. In this hackathon, we demonstrated the usefulness of testing and developed a set of recommendations that should improve the development of tests. We also demonstrated the feasibility of producing test suites for already-established microbial bioinformatics software (Table S1).

## Supplementary Data

Supplementary material 1
