# Supplementary material for: Software testing in microbial bioinformatics: a call to action
Source: Microb Genom. 2022 Mar 8;8(3):000790. doi: 10.1099/mgen.0.000790 (PMC9176277; doi:10.1099/mgen.0.000790)
Supplement: Supplementary material 1 [file mgen-8-0790-s001.pdf]

```
# This is a basic workflow to help you get started with Actions
name: softwareX

# This controls when the action will be triggered.
on:
  push:
    branches: [ main, dev ]
  pull_request:
    branches: [ main, dev ]

# A workflow run is made up of one or more jobs that can run sequentially or in parallel
jobs:
  # This workflow contains a single job called "build"
  build:
    # The type of runner that the job will run on
    runs-on: ${{ matrix.os }}
    strategy:
      matrix:
        os: ["ubuntu-latest", "macos-latest"]
        python-version: [3.5, 3.6, 3.7, 3.8]

    # Steps represent a sequence of tasks that will be executed as part of the job
    steps:
      # Checks-out your repository under $GITHUB_WORKSPACE, so your job can access it
      - uses: actions/checkout@v2
        with:
          path: softwareX
      - name: Set up Python ${{ matrix.python-version }}
        uses: actions/setup-python@v2
        with:
          python-version: ${{ matrix.python-version }}
      # Runs a single command using the runners shell
      - name: Run a one-line script
        run: echo Hello, world!
      # Run test suite if included in the software
      - name: Run test suite
        run: |
          softwareX --test
      # Alternatively, run manual tests
      - name: Run annotation test
        run: |
          softwareX --input test/test.fna --output test_out.gff
          cmp test_out.gff test/result.gff
```

This workflow is named “softwareX”

This workflow will be triggered by pushes or pull requests on the main and dev branches

In GitHub Actions, one can easily define matrices which can also be combined. This workflow runs tests using combined matrices of operating system and Python versions (testing a total of eight combinations in this example)

On GitHub Marketplace, Actions from other developers are available. These can be used to perform common tasks, such as checkout a GitHub repository or setup a particular version of Python.

The “run” keyword specifies commands that are run. These can be single lines or multiple lines. If a command in a job exits with an error, the job will fail.

In this example, a test suite included in the software is run (typically invoked by using the flag “--test”).

Here, a small FASTA file is annotated. Output is compared to an existing output file using “cmp”, which throws an error if files are different.

Figure S1. Example YAML file for a GitHub Actions workflow.

Table S1. Software tested during the ASM NGS 2020 hackathon

Software Name	Software (URL)	Test File (URL)	Literature Citation (DOI)
**BUSCO**	https://gitlab.com/ezlab/busco	https://github.com/microbinfie-hackathon2020/CSIS/blob/main/.github/workflows/busco.yml	10.1093/bioinformatics/btv351
**Centrifuge**	https://github.com/DaehwanKimLab/centrifuge	https://github.com/microbinfie-hackathon2020/CSIS/blob/main/.github/workflows/centrifuge.yml	10.1101/gr.210641.116
**CheckM**	https://github.com/Ecogenomics/CheckM	https://github.com/microbinfie-hackathon2020/CSIS/blob/main/.github/workflows/checkm.yml	10.1101/gr.186072.114
**chewBBACA**	https://github.com/B-UMMI/chewBBACA	https://github.com/B-UMMI/chewBBACA/blob/master/.github/workflows/chewbbaca.yml	10.1099/mgen.0.000166
**CSIS**	https://github.com/microbinfie-hackathon2020/CSIS	https://github.com/microbinfie-hackathon2020/CSIS/blob/main/.github/workflows/CSIS.yml	this manuscript
**Genotyphi**	https://github.com/katholt/genotyphi	https://github.com/microbinfie-hackathon2020/CSIS/blob/main/.github/workflows/genotyphi.yml	10.1038/ncomms12827

**Kraken**	https://github.com/DerrickWood/kraken	https://github.com/microbinfie-hackathon2020/CSIS/blob/main/.github/workflows/kraken.yml	10.1186/gb-2014-15-3-r46
**Kraken2**	https://github.com/DerrickWood/kraken2	https://github.com/microbinfie-hackathon2020/CSIS/blob/main/.github/workflows/kraken2.yml	10.1186/s13059-019-1891-0
**KrakenUniq**	https://github.com/fbreitwieser/krakenuniq	https://github.com/microbinfie-hackathon2020/CSIS/blob/main/.github/workflows/krakenuniq.yml	10.1186/s13059-018-1568-0
**Pangolin**	https://github.com/cov-lineages/pangolin	https://github.com/microbinfie-hackathon2020/CSIS/blob/main/.github/workflows/pangolin.yml	absent
**Prokka**	https://github.com/tseemann/prokka	https://github.com/microbinfie-hackathon2020/CSIS/blob/main/.github/workflows/prokka.yml	10.1093/bioinformatics/btu153
**Quast**	https://github.com/ablab/quast	https://github.com/microbinfie-hackathon2020/CSIS/blob/main/.github/workflows/quast.yml	10.1093/bioinformatics/btt086
**Shovill**	https://github.com/tseemann/shovill	https://github.com/microbinfie-hackathon2020/CSIS/blob/main/.github/workflows/shovill.yml	absent
**SKESA**	https://github.com/ncbi/SKESA	https://github.com/microbinfie-hackathon2020/CSIS/blob/main/.github/workflows/skesa.yml	10.1186/s13059-018-1540-z

**Trycycler**	https://github.com/rrwick/Trycycler	https://github.com/microbinfie-hackathon2020/CSIS/blob/main/.github/workflows/trycycler.yml	10.5281/zenodo.4430941
**Unicycler**	https://github.com/rrwick/Unicycler	https://github.com/microbinfie-hackathon2020/CSIS/blob/main/.github/workflows/unicycler.yml	10.1371/journal.pcbi.1005595
